# Translucency evaluation of an ultra-translucent monolithic zirconia and a super translucent multilayer zirconia: a pilot study on the thickness effect

**DOI:** 10.1080/07853890.2021.1897400

**Published:** 2021-09-28

**Authors:** Ana Filipa Pereira, Luís Proença, Alexandra Pinto, Inês Carpinteiro, Ana Cristina Azul, Inês Caldeira Fernandes

**Affiliations:** a Instituto Universitário Egas Moniz (IUEM), Egas Moniz Cooperativa de Ensino Superior, Caparica, Portugal; b Centro de Investigação Interdisciplinar Egas Moniz (CiiEM), Egas Moniz Cooperativa de Ensino Superior, Caparica, Portugal

## Abstract

**Introduction:**

The increasing application of zirconia as an aesthetic material has led to an evolution with improvement on its optical properties [1]. Nowadays, zirconia presents a compatible translucency with natural teeth. Depending on the clinical situation, you can choose a more translucent or more opaque zirconia. Due to its excellent mechanical properties, it is possible to return the function and aesthetics through a restoration with thicknesses lower than those required by other materials [2].

**Materials and methods:**

Twenty specimens of pre-sintered ultra-translucent (UT) monolithic zirconia (Bloomden W00098014UT) and twenty specimens of pre-sintered super translucent (ST) multilayer zirconia (Bloomden W00098016ST- ML-A2) were cut through the computer-aided design/computer-aided manufacturing system (Wieland Dental). For each type of zirconia, four subgroups (*n* = 5) were defined according to its thickness: 0.5, 1.0, 1.5 and 2.0 mm. All monolithic specimens were coloured with colouring liquid (BloomZir® UT Coping Crown A2) for 2 min. Finally, all zirconia specimens were sintered in the furnace (IMES-Wieland Zeno^®^ Fire) at a temperature range of 1500 − 1550 °C and subsequently submitted to an ultrasonic bath (VGT-2120QTD 20 L) [3]. The values of L*, a* and b* were measured, under natural light (D65), using the spectrophotometer SpectroShade Micro (MHT S.p.A., Arbizzano di Negrar, Itália) at six different locations on a white background (*Comission Internationale de l’ Éclairage* (CIE) L*=95.6 a*= 0.8, b*= 0.1) and on a black background ((CIE) L *= 13.2, a *= 0.8, b *= −0.7). Translucency was assessed through the contrast ratio (CR) [4] and the translucency parameter (TP) [5]. Data was submitted to descriptive statistical analysis.

**Results:**

Mean CR values ranged from 0.7 (±0.0) to 0.9 (±0.0) in UT monolithic zirconia and 0.8 (±0.0) to 0.9 (±0.0) in the ST multilayer zirconia. Mean TP values ranged from 14.1 (±0.3) to 26.7 (±0.4) in UT monolithic zirconia and 9.9 (±0.4) to 18.6 (±0.3) in ST multilayer zirconia. As thickness increases, translucency values decreased, in both materials.

**Discussion and conclusions:**

Overall, ultra-translucent monolithic zirconia was found to be more translucent than the super translucent multilayer zirconia. The translucency showed to decrease when zirconia thickness increases.
